# Supplementary material for: Interprofessional Therapeutic Drug Monitoring of Carbapenems Improves ICU Care and Guideline Adherence in Acute-on-Chronic Liver Failure
Source: Antibiotics (Basel). 2023 Dec 14;12(12):1730. doi: 10.3390/antibiotics12121730 (PMC10740747; doi:10.3390/antibiotics12121730)
Supplement: Supplementary file 1 [file antibiotics-12-01730-s001.zip › antibiotics-2769478-supplementary.pdf]

Supplementary Table S1. Presentation of patient characteristics, including sex and age, and initial, 2nd, and 3rd results of therapeutic drug monitoring (TDM) for meropenem (meropenem serum concentration in mg/l), as well as initial, 2nd, and 3rd recommendation of the interprofessional team.

Patient	Sex	Age	Initial TDM	Initial Recommendation	2nd TDM	2nd Recommendation	3rd TDM	3rd Recommendation
1	M	36	10.0	No change in meropenem dosage	24.4	Decrease in meropenem dosage	13.5	No change in meropenem dosage
2	M	52	8.4	No change in meropenem dosage	-/-	-/-	-/-	-/-
3	M	40	11.4	No change in meropenem dosage	24.6	Change to another antibiotic therapy	-/-	-/-
4	F	34	32.8	Decrease in meropenem dosage	-/-	-/-	-/-	
5	M	58	20.5	Change to another antibiotic therapy	-/-	-/-	-/-	-/-
6	M	74	33.2	Decrease in meropenem dosage	-/-	-/-	-/-	-/-
7	F	68	18.1	No change in meropenem dosage due to improving kidney function	14.2	Stopping of antibiotic therapy	-/-	-/-
8	M	62	21.0	Change to another antibiotic therapy	-/-	-/-	-/-	-/-
9	F	66	13.7	No change in meropenem dosage	14.9	No change in meropenem dosage	-/-	-/-
10	M	41	16.7	Decrease in meropenem dosage	9.5	No change in meropenem dosage	-/-	-/-
11	F	69	35.2	Decrease in meropenem dosage	-/-	-/-	-/-	-/-
12	M	54	13.3	No change in meropenem dosage	9.9	No change in meropenem dosage	-/-	-/-
13	M	59	14.7	No change in meropenem dosage	-/-	-/-	-/-	-/-
14	F	70	8.8	No change in meropenem dosage	19.3	Change to another antibiotic therapy	-/-	-/-
15	M	65	39.0	Decrease in meropenem dosage	11.8	No change in meropenem dosage	-/-	-/-
16	M	58	10.0	No change in meropenem dosage	-/-	-/-	-/-	-/-
17	F	67	35.5	Change to another antibiotic therapy	-/-	-/-	-/-	-/-
18	M	40	14.0	No change in meropenem dosage	-/-	-/-	-/-	-/-
19	M	55	19.7	Decrease in meropenem dosage	10.5	No change in meropenem dosage	-/-	-/-
20	F	36	29.5	Decrease in meropenem dosage	20.5	No change in meropenem dosage due to initiation of dialysis	-/-	-/-
21	M	69	31.1	Decrease in meropenem dosage	13.8	Change to another antibiotic therapy	-/-	-/-
22	M	52	26.9	Decrease in meropenem dosage	-/-	-/-	-/-	-/-
23	F	66	13.1	Change to another antibiotic therapy	-/-	-/-	-/-	-/-
24	M	53	18.8	Change to another antibiotic therapy	-/-	-/-	-/-	-/-
25	M	62	27.5	Decrease in meropenem dosage	9.0	No change in meropenem dosage	10.2	No change in meropenem dosage
